# Extracellular vesicles in metabolic disease

**DOI:** 10.1007/s00125-019-05014-5

**Published:** 2019-11-05

**Authors:** Naveed Akbar, Valerio Azzimato, Robin P. Choudhury, Myriam Aouadi

**Affiliations:** 1 Division of Cardiovascular Medicine, Radcliffe Department of Medicine, University of Oxford, Level 6, West Wing, John Radcliffe Hospital, Oxford, OX3 9DU UK; 2 Integrated Cardio Metabolic Centre, Department of Medicine, Karolinska Institutet, NOVUM, Blickagången 6, 141 57 Huddinge, Sweden

**Keywords:** Adipocytes, Diabetes, Diagnostic, Endothelial cells, Exosomes, Extracellular vesicle, Immune cells, Macrophages, Metabolic dysfunction, Platelets, Review, Therapeutic

## Abstract

Extracellular vesicles (EVs) are submicron-sized lipid envelopes that are produced and released from a parent cell and can be taken up by a recipient cell. EVs are capable of mediating cellular signalling by carrying nucleic acids, proteins, lipids and cellular metabolites between cells and organs. Metabolic dysfunction is associated with changes in plasma concentrations of EVs as well as alterations in their EV cargo. Since EVs can act as messengers between parent and recipient cells, they could be involved in cell-to-cell and organ-to-organ communication in metabolic diseases. Recent literature has shown that EVs are produced by cells within metabolic tissues, such as adipose tissue, pancreas, muscle and liver. These vesicles have therefore been proposed as a novel intercellular communication mode in systemic metabolic regulation. In this review, we will describe and discuss the current literature that investigates the role of adipose-derived EVs in the regulation of obesity-associated metabolic disease. We will particularly focus on the EV-dependent communication between adipocytes, the vasculature and immune cells in type 2 diabetes.

**Electronic supplementary material:**

The online version of this article (10.1007/s00125-019-05014-5) contains a slide of the figure for download, which is available to authorised users.

## Introduction

### Metabolic disease


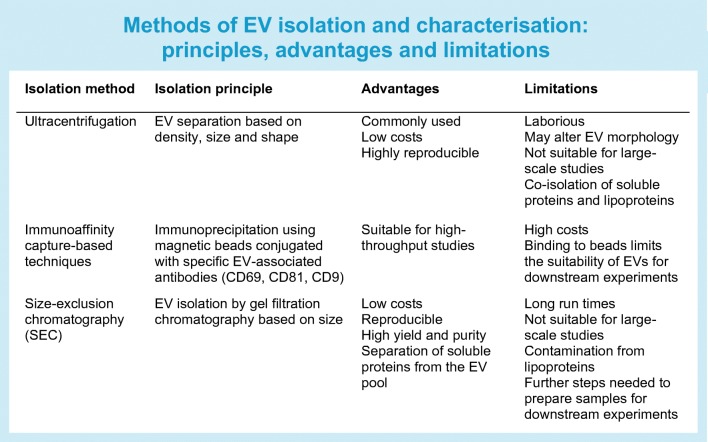

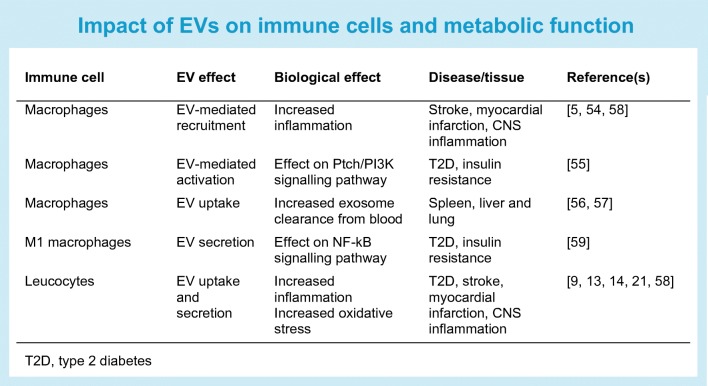
Metabolic dysfunction is a collective term for the clustering of disease risk factors, including hyperglycaemia, dyslipidaemia, hypertension, obesity and insulin resistance. Metabolic dysfunction significantly increases disease risk for cardiovascular diseases such as acute myocardial infarction and stroke. The combined pathogenesis of metabolic dysfunction implicates numerous cell types, tissues, organs, inflammatory signalling cascades and humoral factors. Blood-based biomarkers, such as glucose, insulin and HbA_1c_ are used in the diagnosis and management of type 2 diabetes and plasma lipoproteins in cardiovascular diseases. Other blood-borne factors, such as inflammatory cytokines (IL-6, TNF-α, IL-10), oxidised low-density lipoproteins, triacylglycerols, leptin, gherlin and adiponectin are imperfect markers of metabolic disease [1] but are related to measures of insulin resistance, visceral adiposity and atherosclerotic disease. The identification of plasma molecules that serve as prognostic and diagnostic biomarkers for metabolic disease and its pathological complications remains challenging because the factors listed above provide little insight into the tissue types and organs afflicted by metabolic dysfunction.

### Extracellular vesicles

Extracellular vesicles (EVs) are membrane-enclosed envelopes that carry lipids, proteins, nucleic acids (DNA, mRNA, microRNA and long non-coding RNAs) and metabolites. EVs can bear features (RNAs, DNA and lipids) of their parent cell of origin and may provide diagnostic and prognostic value in metabolic dysfunction [2–12]. They can mediate communication locally within tissues between cells of the same origin, i.e. endothelial cells, hepatocytes, immune or pancreatic cells, and cross organ systems through liberation into peripheral blood [13, 14]. EVs thus have potential as biomarkers in the prognosis and diagnosis of metabolic disease [4, 6, 15, 16].

**Fig. 1:**
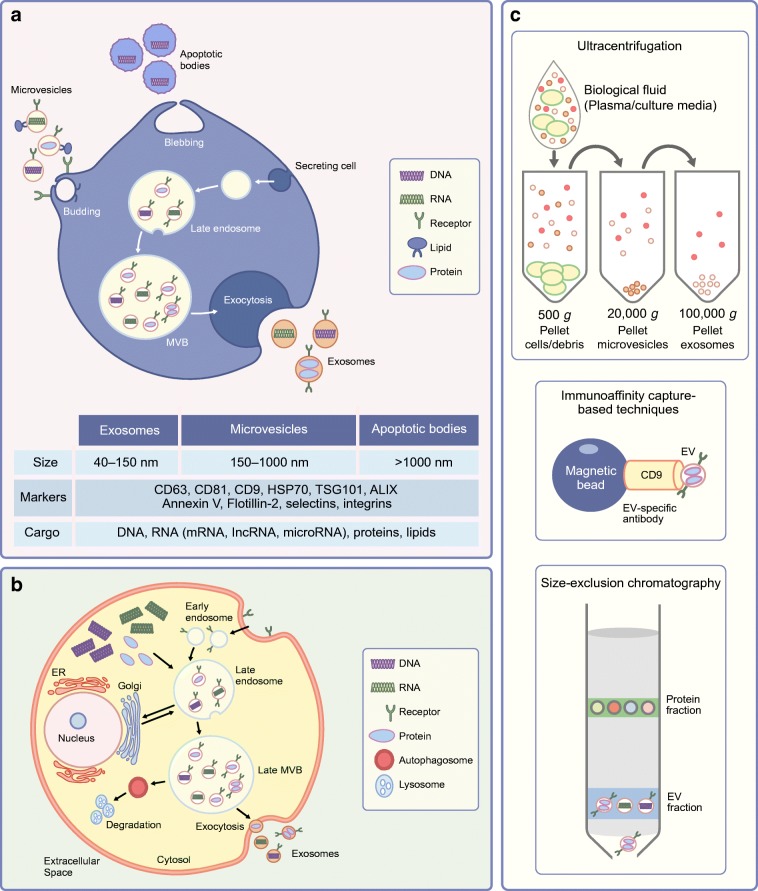
The EV landscape at a glance from biogenesis to isolation and characterisation. (**a**) A schematic representation of the cellular origins of EV sub-populations, their respective classifications based on EV size, and associated markers. Apoptotic bodies are generated by apoptotic blebbing and microvesicles by budding of the plasma membrane. Exosomes are generated from late endosomal MVBs, which fuse with the plasma membrane and release small exosomes. ALIX, ALG-2-interacting *protein* X; HSP70, heat shock protein 70; TSG101, tumour susceptibility gene 101. (**b**) The stages of exosome biogenesis. Late endosomes mature into MVBs, which are targeted for degradation or fuse with the plasma membrane to release exosomes. ER, endoplasmic reticulum. (**c**) The most commonly used techniques for EV isolation from biological fluids: ultracentrifugation, immunoaffinity capture and size-exclusion chromatography. This figure is available as a downloadable slide

The term EVs denotes numerous subpopulations of biological vesicles, enclosed with a lipid bilayer. The inward budding of endosomal membranes generates intraluminal vesicles in cytosolic multivesicular bodies (MVBs) and the fusion of these MVBs with the plasma membrane releases so-called small exosomes (40–150 nm). The direct blebbing of EVs from the plasma membrane produces microvesicles (150–1000 nm) and larger apoptotic bodies (>1000 nm) are released by cellular death (Fig. 1). Exosomes, microvesicles and apoptotic bodies are characterised primarily by their size, isolation method and protein cargo, such as the presence of tetraspanins (CD63, CD81, CD9), ALG-2-interacting *protein* X (ALIX), tumour susceptibility gene 101 (TSG101), heat shock protein 70 (HSP70) and annexin V, although discrepancies exist and there is significant overlap between these individual subpopulations [17] (Fig. 1). EVs can be isolated from plasma, urine, cerebral spinal fluid, lymph and conditioned media from cell culture by differential ultracentrifugation. EV preparations can be positive for the EV-associated tetraspanins, TSG101, ALIX and display circular morphology, as determined by transmission electron microscopy. EV preparations may be contaminated by soluble factors, such as proteins and apolipoproteins (Apo-1/2, Apo-B, Apo-B100), cellular components such as nuclear fragments, which can be detected by the presence of histones (H3) and mitochondria, by ATP synthase F1 subunit α (ATP5A)/translocase of outer mitochondrial membrane 20 (TOMM20). EV surface proteins, such as intracellular adhesion molecule-1 (ICAM-1) and vascular cell adhesion molecule-1 (VCAM-1), allow EVs to target and bind to recipient cells through receptor interactions with leucocyte function-associated antigen-1 (LFA-1) and very late antigen-4 (VLA-4), respectively [13, 18]. EV uptake by cells can be mediated by endocytosis (clathrin-, caveolin- and/or lipid-mediated), phagocytosis and micropinocytosis. EVs can also undergo degradation by matrix metalloproteinase at the cell surface and the constituent parts of EVs, such as proteins, RNA and/or lipids, may mediate cellular signalling by receptor binding. Precisely how EV biogenesis and the mechanism of EV uptake are altered in metabolic dysfunction remains unknown. A better understanding of how EVs promote signalling and uptake by cells in metabolic disease may allow the development of novel strategies for EV-targeting, to perturb pathogenic signalling. EVs transport many constituent parts of their parent cells, and methods for EV cargo detection require robust optimisation because there are often limited amounts of starting material available in clinical and rodent studies.

The International Society for Extracellular Vesicles (ISEV) publishes guidelines for the isolation, characterisation and experimental use of EVs [19]. The most popular method for EV isolation remains ultracentrifugation, as it is easily accessible, cheap and requires little prior specialist knowledge or equipment. Ultracentrifugation can co-isolate soluble proteins (cytokines, chemokines and growth factors) and lipoproteins, which carry EV-associated cargo such as miRNAs. Washing of isolated EVs can reduce the level of contamination by soluble proteins and lipoproteins, but ultracentrifugation speeds can alter the morphology of EVs, possibly affecting their biological activity. Immunoaffinity capture using EV-associated antibodies (CD63, CD81 and CD9) conjugated to magnetic beads allows high-throughput processing of clinical samples but is biased towards the isolation of tetraspanin-expressing EVs [20]. Immunoaffinity capture has the added difficulty of binding EV to beads and limits the utilisation of isolated EV for downstream functional characterisation such as in vivo studies. Size exclusion chromatography (SEC) of EVs from biological fluids allows soluble proteins to be separated from the EV pool but does not eliminate contamination with lipoproteins, and SEC EV samples often need to be further concentrated for additional EV analysis, such as proteomics [21] (see Text box: Methods of EV isolation and characterisation: principles, advantages and limitations). EV phenotyping in metabolic disease has revealed a number of dysregulated EV proteins and EV miRNAs from numerous tissue types and plasma from individuals with different types of metabolic dysfunction. This review focuses on the discoveries and descriptions of EV in metabolic disease and the challenges faced to produce an effective EV diagnostic platform for metabolic disease phenotyping. Here we discuss type 2 diabetes and insulin resistance but systematic studies investigating individual cell populations to determine their EV characteristics will enhance the utility of EVs as disease biomarkers and facilitate the creation of EV-based therapeutics.

## EVs as biomarkers of metabolic disease

Patient plasma and urine are rich sources of EVs and studies have demonstrated EV miRNAs as diagnostic tools in individuals suffering from metabolic [2–4, 9] and cardiovascular disease [22, 23]. A recent meta-analysis by Li et al. [24] reported that levels of EVs of platelet, endothelial cell and monocyte origin were significantly raised in individuals with type 2 diabetes from 48 independent studies [24, 25].

EV number correlates positively with systolic and diastolic blood pressure, BMI, HOMA-IR, and HOMA-B (a measure of beta cell function) [6], which are altered in metabolic disease. Circulating levels of plasma EVs are higher in men than in women with and without metabolic disease and are elevated in individuals with impaired glucose tolerance. Obese patients have significantly greater levels of circulating plasma EVs than normal healthy controls and reductions in plasma EVs can be achieved by a low-energy diet, diet plus exercise and weight loss following gastrectomy [26]. Below we summarise key findings in endothelial cell-, platelet-, adipocyte- and monocyte-derived EVs, which are among the most-widely studied EV populations in metabolic disease.

### Platelet-derived EVs

Platelet-derived EVs are primarily characterised by the presence of CD41, CD42b and phosphatidylserine and have been demonstrated to play roles in exercise [27, 28], acute injury [29] and diabetes [30]. Platelet-derived EVs are regarded as the most abundant source of plasma EVs; however, it is unclear as to what proportion of the EV pool they represent, with approximations varying from 50 to 90% [29]. Platelet EV release is calcium dependent and relies on actin cytoskeleton destabilisation [31]. The number of platelet-derived EVs is increased in obese individuals but is not further influenced by the presence of metabolic disease [32]. A better understanding is needed of which metabolic disease factors, i.e. endothelial dysfunction, hypertension and/or dyslipidaemia, alter the properties of platelet-derived EVs in metabolic dysfunction. This will allow the development of diagnostic tests and therapies that target platelet EVs. Individuals with metabolic disease are often prescribed a variety of therapeutic agents, which may influence the number and composition of platelet-derived EVs in patients [33]. Importantly, those that target calcium signalling would alter the biogenesis of platelet-derived EVs.

### Adipocyte-derived EVs

Adipose tissue has been described as a major source of circulating exosomal miRNA by studies using an adipose-tissue-specific knockout of the miRNA-processing enzyme Dicer (AdicerKO) and other studies of samples obtained from humans with lipodystrophy [34, 35]. Adipocyte hypertrophy increases the risk for developing type 2 diabetes [36]. It has also been associated with the elevated production and release of EVs, characterised by the expression of perilipin A [11, 37]. Consistent with these studies, circulating levels of adipocyte-derived EVs have been shown to be increased in obese mice and humans and decreased following energy restriction or bariatric surgery [11, 38]. Interestingly, Hubal et al. have identified 55 adipocyte-derived exosomal miRNAs that are differentially expressed between obese and lean individuals, suggesting that in addition to their higher circulating levels, the exosomal cargo of EVs is regulated in obese individuals [38]. Although the precise mechanism whereby adipose tissue-derived EV production is regulated in obesity is not clear, the adiponectin/T-cadherin system has been implicated in exosome biogenesis and secretion [39].

Recent studies investigating the role of EVs in the regulation of systemic metabolism have identified adipocyte-derived EVs as mediators linking adiposity and insulin resistance in peripheral tissues, such as the liver [40, 41]. EVs can act as messengers between adipocytes and other cells in the adipose tissue. For example, adipocyte-derived EVs can chemoattract monocytes and could therefore contribute to the adipose tissue inflammation observed in obese insulin-resistant animals and humans [37, 42]. EVs could also be transferred from adipose tissue endothelial cells to adipocytes and vice versa in vivo [43]. This transfer event is affected by nutritional and obesity status. While the effect of adipose-derived EVs on metabolism seems to be dependent on the physiological context, EVs from adipose-derived stem cells might have a beneficial effect on insulin sensitivity through their ability to reduce inflammation and induce beigeing in the adipose tissue [44].

Taken together, these studies offer new insights into the complex signalling mechanisms that exist between adipocytes, stromal vascular cells and distal organs in health and metabolic diseases. Further studies are needed to elucidate the precise content of adipose-derived EVs and their impact on surrounding cells and the function of metabolic organs, including different fat depots.

### Adipose tissue macrophage-derived EVs

Obesity and insulin resistance are associated with the accumulation of macrophages in the adipose tissue [45]. Extensive literature has described a detrimental role of adipose tissue macrophages (ATMs) in the regulation of whole body metabolism [46] through excessive production of inflammatory cytokines able to block insulin signalling. Recently exosomes derived from ATMs have been shown to be important mediators in the regulation of adipose tissue function and insulin sensitivity [47]. In obese mice, ATMs secrete miRNA-containing exosomes, which cause glucose intolerance and insulin resistance when administered to lean mice. Conversely, ATM exosomes obtained from lean mice improve glucose tolerance and insulin sensitivity when administered to obese recipients. miRNA-155, one of the miRNAs overexpressed in exosomes derived from obese ATMs, was shown to regulate adipose tissue homeostasis through direct suppression of its targets, the adipogenic transcription factors peroxisome proliferator-activated receptor γ (PPARγ) and CCAAT/enhancer-binding protein β (CEBPβ) [48]. It has also been demonstrated that proinflammatory macrophages preferentially produce miR-155 [49]. These studies highlight not only the importance of macrophages as a source of adipose-exosomes, but also suggest that ATMs could produce EVs containing different types of cargo depending on their phenotype. Since multiple populations of macrophages co-exist within the adipose tissue [36, 50], it would be important to characterise the types of EVs produced by these different populations in health and metabolic diseases.

### Endothelial cell-derived EVs

Endothelial dysfunction is an early vascular abnormality during insulin resistance and is present in metabolic disease. Endothelial cells interface with multiple tissue microenvironments release signals to attract immune cells and capture them from the circulation. Endothelial cell-derived EVs from individuals with metabolic disease have been characterised by the presence of markers CD62E, CD144, CD31, VCAM-1, ICAM-1 and miRNAs-126-3/5p [51]. People with diabetes have lower levels of miRNA-126 and miRNA-26a; hyperglycaemia lowers levels of these two miRNAs in endothelial cells [52, 53]. Hyperglycaemia and endothelial cell activation alter endothelial cell-derived EV mRNAs for inflammatory IL-1β, chemokine ligand 2 (CCL2), VCAM-1 and EV proteins by increasing levels of EV ICAM-1, TNF and TNFα-induced protein 3 (TNFAIP3) [54]. Endothelial cells can transfer EV proteins, such as caveolin-1, and sphingolipids to neighbouring adipocytes during nutrient deficiency [43]. Insulin can influence the proteomic profile of EVs [55] but whether RNA, lipoproteins and proteins from EVs change synergistically during hyperglycaemia or in response to insulin or obesity remains unknown. The number of circulating plasma EVs decreases with advancing age, and EVs from older individuals are more readily internalised by immune cells than EVs from younger individuals [10]. However, which ageing-associated changes influence the pathological features of EVs in the presence of metabolic disease remains ambiguous.

### Metabolic disease: influence of EVs on metabolic tissues and immune cells

Liberated EVs in plasma can be captured by tissue resident populations of macrophages in the liver, marginal zone CD169^+^ macrophages in the spleen and endothelial cells in the lung [56, 57]. EVs from individuals with metabolic disease can mediate differential effects on immune cells and show alterations in their protein cargo [5]. Inflammation [13, 14, 21, 55, 58] and insulin resistance increases EV secretion [9] and EV uptake by leucocytes [9, 13, 14, 21] (see Text box: Impact of EVs on immune cells and metabolic function). In type 2 diabetes, EVs containing sonic hedgehog protein can mediate macrophage activation and insulin resistance [59], although the role of other immune cell-derived EVs, such as those from neutrophils in response to hyperglycaemia, insulin, oxidised LDL, and their role in metabolic homeostasis requires further attention. However, whether these dysregulated EV miRNAs remain altered in macrophage EVs following diet or exercise interventions remains unknown.

Macrophages can be broadly split into proinflammatory M1 or anti-inflammatory M2 phenotypes, according to their activating stimulus, gene expression, metabolic profiles and cellular functions. EVs from inflammatory M1 but not anti-inflammatory M2 macrophages influence adipocyte differentiation and insulin signalling via NF-kB activation and M2 macrophage-derived EVs enhance glucose uptake by adipocytes [60]. Macrophages are highly sensitive to the microenvironment, and how altered tissue homeostasis impacts individual niches of tissue-resident macrophages mediate EV uptake and EV release may provide insight into why some tissues are more prone to metabolic stress and dysfunction.

### Gut microbiota-derived EVs

The gut microbiota can generate EVs [61] to alter insulin sensitivity by impairing glucose metabolism in skeletal muscle [62] and disrupt circadian biology to mediate metabolic dysfunction and inflammatory cell recruitment [7]. The gut microbiome is altered in individuals with metabolic disease but the extent to which the gut microbiota contribute to the pathogenesis of metabolic disease via the action of EVs remains uncertain. Dietary EVs can alter the gut microbiome, for example, bovine milk exosomes significantly alter populations of Lachnospiraceae, Ruminococcaceae and the Verrucomicrobiaceae families in non-bovine species [63, 64], and bovine milk EVs alter amino acid metabolism in the liver [65]. Dietary and gut microbe EVs may work synergistically to promote the pathogenesis of metabolic disease, and stool-derived EVs may be a useful biomarker for alterations in metabolic homeostasis. Exactly which individual populations of gut microbes produce and release EVs that can influence metabolic homeostasis by altering digestion and/or nutrient absorption requires further investigation and may provide insight into how dietary interventions mediate beneficial effects for individuals with metabolic disease.

### Influence of drugs for metabolic disease on EVs

A significant proportion of patients with conditions that fall under the umbrella of metabolic disease are prescribed a range of therapeutic interventions, including statins, anti-hypertensive medications, metformin, sulfonylureas and sodium–glucose cotransporter 2 (SGLT2) inhibitors. There is limited information available on the effect of metabolic drugs on the generation, release and cargo of EVs on mammalian biology and on host gut microbiomes. Determination of whether EV biogenesis and cargo are altered by widely prescribed medicine would allow delineation of EVs as a consequence of therapeutic intervention and a possible platform for disease monitoring.

## Discussion and conclusions

Metabolic investigations have used a range of mammalian cells to study EVs subpopulations but there is a lack of systematic in-depth characterisation of all the EV populations in single cell types from multiple sources, i.e. endothelial cells or tissue resident macrophages from different organs. Identification of differences between EVs from the same cell type in different microenvironments may allow better differentiation of pathological EV-signals for disease phenotyping in peripheral blood. Furthermore, there is limited information on how EV biogenesis pathways are implicated in metabolic disease and how these are altered to influence the release of EV and their cargo in individuals with metabolic disease. A better understanding of how EV biogenesis pathways are dysregulated in metabolic disease may allow the generation of targeted therapies to perturb EV signalling and the development of new EV diagnostic tests.

EVs protect miRNA from nucleases in the peripheral blood. Other non-coding RNAs, such as lncRNAs are enriched in EVs but knowledge of their roles in cellular response and metabolic disease homeostasis is limited. It is currently difficult to ascertain whether EVs, especially those from clinical cohorts are solely biomarkers of existing metabolic disease or triggered by disturbances in homeostasis, such as insulin insensitivity and hyperglycaemia. By utilising primary human cell cultures in addition to clinical cohorts, researchers can begin to understand whether metabolic stimuli such as hyperglycaemia are inducing transient changes in EVs or alter cellular function that can be detected in EV cargo.

Metabolic EV studies to date have largely focused on either EV miRNA or EV proteome but few efforts have been made to explore synergistic/co-ordinated disease signatures in the combined EV RNA–proteome. This combined approach would support the notion that EVs are generated under specific disease conditions and promote pathology by direct cellular targeting. The pool of EVs in plasma and urine offers opportunities for disease phenotyping. However, research efforts are hindered by an inability to definitely ascertain the tissues and parent cells producing and releasing EV. Combined ‘-omics’ approaches for EV phenotyping would provide vital insight into the characteristics of EVs in metabolic disease and potentially identify new strategies for disease treatment.

## Electronic supplementary material

ESM 1 (PPTX 164 kb)

## Abbreviations

ATM: Adipose tissue macrophages
EV: Extracellular vesicle
ICAM-1: Intracellular adhesion molecule-1
MVB: Multivesicular body
SEC: Size exclusion chromatography
VCAM-1: Vascular cell adhesion molecule-1
