# Supplementary material for: Extracellular vesicles in metabolic disease
Source: Diabetologia. 2019 Nov 5;62(12):2179–87. doi: 10.1007/s00125-019-05014-5 (PMC6861353; doi:10.1007/s00125-019-05014-5)
Supplement: Supplementary file 1 — (PPTX 164 kb) [file 125_2019_5014_MOESM1_ESM.pptx]

## Slide 1
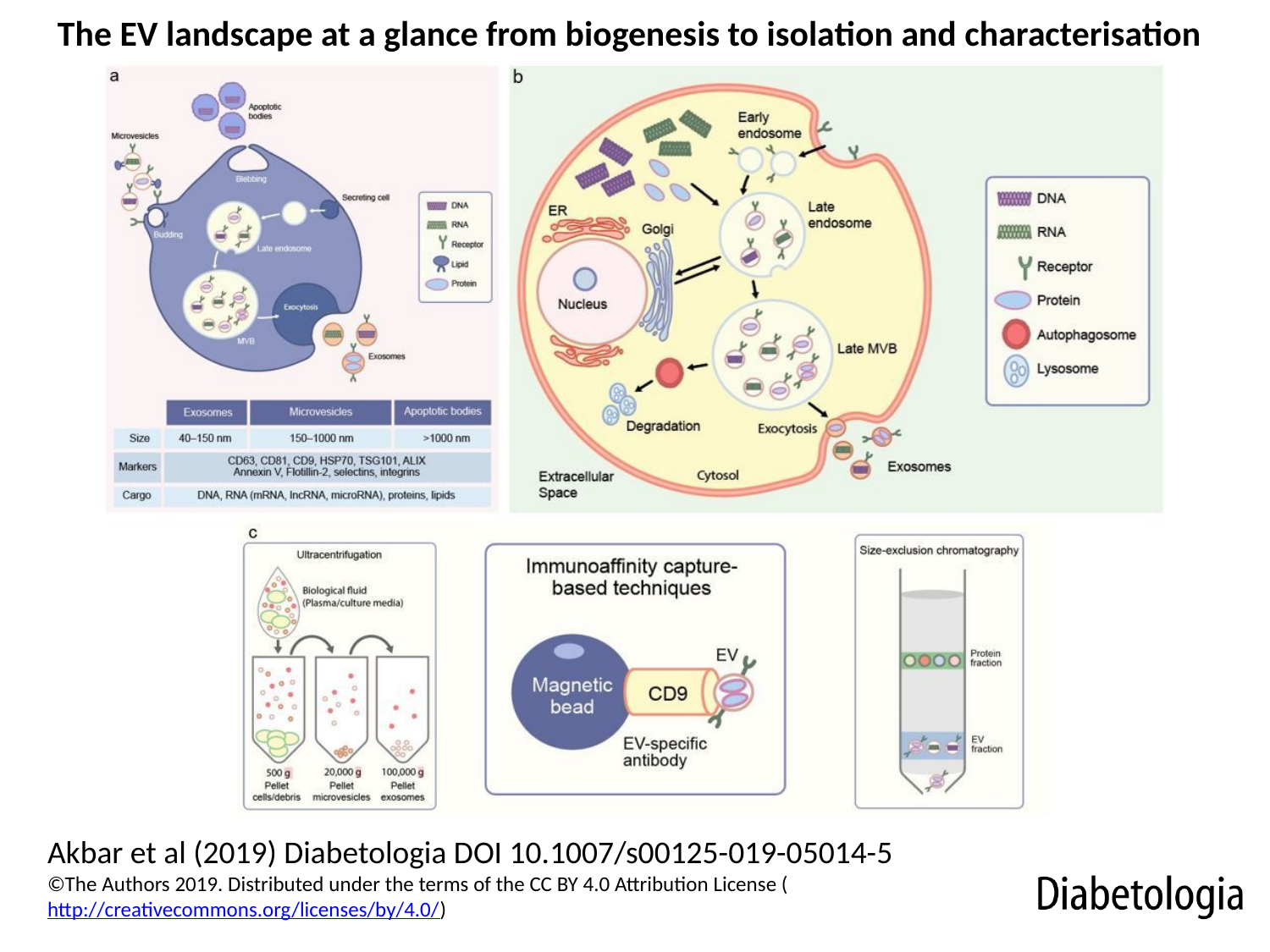

The EV landscape at a glance from biogenesis to isolation and characterisation
b
Akbar et al (2019) Diabetologia DOI 10.1007/s00125-019-05014-5
©The Authors 2019. Distributed under the terms of the CC BY 4.0 Attribution License (http://creativecommons.org/licenses/by/4.0/)
